# Gene Expression Networks in the Murine Pulmonary Myocardium Provide Insight into the Pathobiology of Atrial Fibrillation

**DOI:** 10.1534/g3.117.044651

**Published:** 2017-07-18

**Authors:** Jordan K. Boutilier, Rhonda L. Taylor, Tracy Mann, Elyshia McNamara, Gary J. Hoffman, Jacob Kenny, Rodney J. Dilley, Peter Henry, Grant Morahan, Nigel G. Laing, Kristen J. Nowak

**Affiliations:** * Harry Perkins Institute for Medical Research,; † Centre for Medical Research, University of Western Australia, Queen Elizabeth II Medical Centre, Nedlands, 6009, Australia; ‡ School of Biomedical Sciences, University of Western Australia, Queen Elizabeth II Medical Centre, Nedlands, 6009, Australia; § Ear Sciences Centre, Faculty of Health and Medical Sciences, University of Western Australia, Queen Elizabeth II Medical Centre, Nedlands, 6009, Australia

**Keywords:** pulmonary myocardium, eQTL, atrial fibrillation, gene network

## Abstract

The pulmonary myocardium is a muscular coat surrounding the pulmonary and caval veins. Although its definitive physiological function is unknown, it may have a pathological role as the source of ectopic beats initiating atrial fibrillation. How the pulmonary myocardium gains pacemaker function is not clearly defined, although recent evidence indicates that changed transcriptional gene expression networks are at fault. The gene expression profile of this distinct cell type *in situ* was examined to investigate underlying molecular events that might contribute to atrial fibrillation. Via systems genetics, a whole-lung transcriptome data set from the BXD recombinant inbred mouse resource was analyzed, uncovering a pulmonary cardiomyocyte gene network of 24 transcripts, coordinately regulated by chromosome 1 and 2 loci. Promoter enrichment analysis and interrogation of publicly available ChIP-seq data suggested that transcription of this gene network may be regulated by the concerted activity of NKX2-5, serum response factor, myocyte enhancer factor 2, and also, at a post-transcriptional level, by RNA binding protein motif 20. Gene ontology terms indicate that this gene network overlaps with molecular markers of the stressed heart. Therefore, we propose that perturbed regulation of this gene network might lead to altered calcium handling, myocyte growth, and contractile force contributing to the aberrant electrophysiological properties observed in atrial fibrillation. We reveal novel molecular interactions and pathways representing possible therapeutic targets for atrial fibrillation. In addition, we highlight the utility of recombinant inbred mouse resources in detecting and characterizing gene expression networks of relatively small populations of cells that have a pathological significance.

In mammals, the pulmonary myocardium forms an atriovenous junction where the atrial myocardium extends into the vena cavae and pulmonary veins, forming a sleeve of myocardial tissue around the veins (Nathan and Eliakim 1966). This myocardial sleeve is thought to maintain venous pressure and prevent blood reflux from the atrium during contraction (Nathan and Eliakim 1966). Interestingly, autonomous electrical activity (Brunton and Fayrer 1876) and ectopic beats originating from the pulmonary myocardium (Chen *et al.* 1999) are implicated in atrial fibrillation. This is important because it has been estimated that atrial fibrillation affects >33 million people globally (Chugh *et al.* 2014). Further, atrial fibrillation is associated with stroke, heart failure, significant morbidity, and increased mortality, leading to an estimated annual cost of $6.65 billion in the United States in 2005 (Reynolds and Essebag 2012). A number of studies have attempted to characterize and define the involvement of the pulmonary myocardium in atrial fibrillation (Chen *et al.* 1999; Hassink *et al.* 2003; Kholová and Kautzner, 2003, 2004; Mommersteeg *et al.* 2007; Saito *et al.* 2000; Steiner *et al.* 2006; Ye *et al.* 2015). However, this small population of cells are difficult to analyze *in situ* and have not been extensively studied in the endogenous context.

Analyses of the prevalence, length, and thickness of myocardial extensions has resulted in conflicting reports as to whether various parameters do (Hassink *et al.* 2003; Kholová and Kautzner 2003) or do not (Kholová and Kautzner 2004; Saito *et al.* 2000) correlate with atrial fibrillation. However, mutations in genes encoding the transcription factors *Nkx2-5* (Huang *et al.* 2013; Xie *et al.* 2013), *Pitx2c* (Qiu *et al.* 2014; Wang *et al.* 2014), and *GATA6* (Li *et al.* 2012) are all associated with atrial fibrillation; these genes are known to be required for cardiovascular development. These data suggest that perturbation of the transcriptional program associated with cardiac muscle (but not necessarily disrupting morphology) may underlie the association of the pulmonary myocardium with atrial fibrillation. Consistent with this, both PITX2C and NKX2-5 are required to direct and maintain the identity of the pulmonary myocardium (Mommersteeg *et al.* 2007). Lost or reduced expression of NKX2-5 causes pulmonary myocardium cells to adopt a pacemaker-like phenotype similar to cells of the sinus horn myocardium, without altering cellular morphology (Mommersteeg *et al.* 2007; Ye *et al.* 2015). This implies that healthy pulmonary myocardium could gain pacemaker function from which an ectopic beat might originate via a shift in the gene expression program away from that of working myocardium and toward a pacemaker program. Thus, molecular analysis of pulmonary myocardium cells *in vivo* is warranted and could provide a powerful tool for understanding the pathobiology of atrial fibrillation and identifying novel therapeutic targets. This led us to investigate gene regulatory mechanisms that might drive the expression of cardiac genes in this tissue.

In this study, we used a systems-genetics approach to conduct complex trait analysis and uncover gene networks in the pulmonary myocardium *in situ* (Chesler *et al.* 2005; Sieberts and Schadt 2007). Systems-genetics approaches use genetic reference populations such as the BXD resource, a set of recombinant inbred mice which are the progeny of an intercross between the two parental mouse strains C57BL/6J and DBA/2J (Andreux *et al.* 2012; Peirce *et al.* 2004). Recombinant inbred strains like the BXD and the Collaborative Cross (Ram *et al.* 2014; Threadgill *et al.* 2011) are especially suited for systems-genetics studies because each strain is effectively immortal (Andreux *et al.* 2012). They provide a powerful test bed for linking genetic loci to variation, including mapping loci controlling gene expression levels. Such loci are termed expression quantitative trait loci (eQTL) (Chesler *et al.* 2005). Importantly, analyses of eQTL provide a means for detecting specific regulators of a gene of interest as well as identification of networks of coregulated transcripts (Andreux *et al.* 2012; Hall *et al.* 2014).

We have analyzed a publicly available BXD steady-state, whole-lung transcriptome data set generated from 51 BXD strains (Alberts *et al.* 2011) to interrogate the molecular phenotype of the pulmonary myocardium. Based on the assumption that genes with highly correlated expression in a tissue are likely to act in a common network or biological process, Alberts *et al.* (2011) previously identified gene networks associated with specific B- and T-cell populations from this same whole-lung data set. We reasoned that we could use a similar approach with the lung data set to identify and characterize pulmonary myocardium cells using the cardiac troponin I type 3 (*Tnni3*) gene (previously shown to be expressed in pulmonary myocardium) (Millino *et al.* 2000) by investigating highly correlated transcripts associated with cardiac muscle. This yielded 24 transcripts significantly correlated with the expression of *Tnni3*, which form the basis of a pulmonary myocardium gene network.

## Materials and Methods

### Analysis of public data sets

The GeneNetwork suite of online programs, incorporating WebQTL (Chesler *et al.* 2004; Wang *et al.* 2003), was used to analyze the publicly available BXD lung transcriptome data set (GN160) (www.genenetwork.org/webqtl/main.py) generated by Alberts *et al.* (2011). Genome-wide association analyses were performed using gene expression microarray values measured on a log_2_ scale. Correlation analysis was performed with Pearson’s correlation, and outliers were removed. For interval mapping, likelihood ratio statistic (LRS) scores were plotted on the *y*-axis *vs.* chromosomal location on the *x*-axis (autosomes and X chromosome). An LRS score >17 was significant and an LRS score >10 was suggestive at a genome-wide *P* value of <0.05 using 5000 permutations. LRS scores were computed using Haley–Knott regression (Haley and Knott 1992). The genomic intervals analyzed were selected based on the location at which the eQTL peak crossed the suggested or significant LRS threshold. Principal component analysis (PCA) was used to merge multiple gene expression traits into a single, synthetic trait that represented a cell-specific gene expression signature.

### Functional annotation and enrichment analysis

First-pass assessment of gene function was obtained from the GeneCards database (www.genecards.org) (Safran *et al.* 2010) and a tissue expression profile was analyzed using the Fantom5 database (http://fantom.gsc.riken.jp/zenbu/) (Forrest *et al.* 2014; Severin *et al.* 2014). The top 100 transcripts (mapping to 78 unique genes) that correlated with the pulmonary myocardium PCA trait were submitted for enrichment analyses and gene ontology (GO) using the WEB-based GEne SeT AnaLysis Toolkit (WebGestalt) (Wang *et al.* 2013). This resource incorporates information from different public databases including the Kyoto Encyclopedia of Genes and Genomes (KEGG), WikiPathways, and human phenotype ontology. We also used the oPOSSUM-3 Web-based tool (Kwon *et al.* 2012) to identify overrepresentation of transcription factor binding sites and integrated ChIP-seq data from two previously published data sets (Dupays *et al.* 2015; He *et al.* 2011).

### Immunohistochemistry

Mouse experiments were approved by the University of Western Australia’s Animal Ethics Committee. Four ∼8-wk-old male BALB/c mice were killed with an overdose of pentobarbitone (160 mg/kg via intraperitoneal injection) and fixed by cardiac perfusion with heparinized phosphate buffered saline (PBS) (15 IU/ml; heparin) followed by fixative (2% paraformaldehyde with 0.2% picric acid in PBS, pH 7.4). Heart and lungs were excised and postfixed for 48 hr at 4° in the same fixative. Tissue was washed, dehydrated, and embedded in paraffin wax. Five-micrometer-thick sections through lungs or ventricular myocardium (control) were cut and mounted onto Superfrost Plus microscope slides (Sigma-Aldrich).

The distribution of TNNI3 in BALB/c lung tissue was detected with a mouse monoclonal IgG_2a_ antibody (Abcam, clone 4C2). Briefly, sections were dewaxed, rehydrated, and subjected to 20 min of heat-induced epitope retrieval (HIER) (pressure cooker) in Tris buffered saline (TBS)/EDTA buffer, pH 9.0 (Vector Laboratories). After 15 min cooling at room temperature, sections were washed in TBS (pH 7.4) and incubated in anti-TNNI3 primary antibody or mouse IgG isotype control (2 μg/ml in 3% fish skin gelatin in TBS) overnight at 4°. Sections were then washed in TBS for 1 hr before incubating in Mouse on Mouse Polymer (Abcam) for 30 min, washed in TBS, and incubated with diaminobenzidine (0.4 mg/ml, 3 min). Sections were counterstained with Mayers hematoxylin, blued with Scotts Tap Water Substitute, washed and dehydrated through graded alcohols to xylene, then coverslipped with Depex mounting medium. Cardiac α-actin (ACTC1) was detected with a mouse monoclonal IgG_1_ antibody (Simga-Aldrich, clone Ac1-20.4.2), or mouse isotype control at 5 μg/ml, with the same protocol as above but with HIER in citrate buffer with pH 6.0 (Vector Laboratories). The presence of smooth muscle α-actin (ACTA2) was detected in a similar fashion, using mouse monoclonal IgG_2a_ antibody (Sigma-Aldrich, clone 1A4) at 9 μg/ml, and detection as above, but without HIER. Tissue was permeablized with 1% Triton X-100 for 15 min prior to the application of the primary antibody. Digital images were acquired on an Aperio ScanScope XT digital slide scanner (Leica Technologies). Images are typically of the left lobe and right middle lobe of four mice.

### Data availability

All data used for this project are publicly available and accessible online (GN accession no. GN160).

## Results

### Tnni3 can be used to identify a pulmonary myocardium gene network

**Figure 1:**
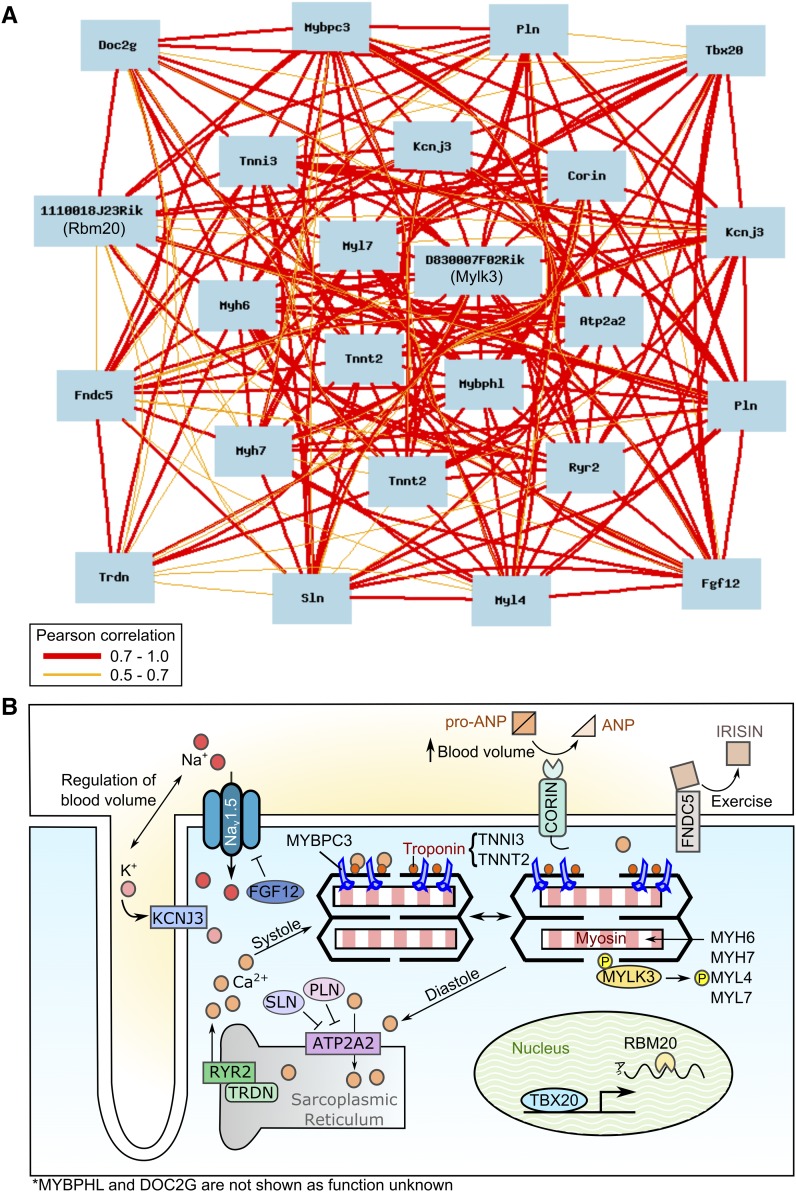
Identification of a pulmonary myocardium gene network. The top 100 transcripts that correlated with *Tnni3* in the BXD whole-lung transcriptome data set were used to generate a network graph. This resulted in a network of 24 transcripts with a Pearson correlation >0.8. (A) These 24 transcripts were subsequently regraphed to interrogate relationships at Pearson correlation values between 0.7 and 1.0 (red lines) and between 0.5 and 0.7 (orange lines). (B) The functional relationships of the 21 proteins translated from these 24 transcripts were also identified. All proteins except DOC2G and MYBPHL had known roles in cardiac function including structural sarcomeric proteins, regulation of sarcomere assembly, ion transport, transcriptional and post-transcriptional regulation, and hormone signaling.

Since the expression of TNNI3 has previously been used as a marker for pulmonary myocardium (Millino *et al.* 2000; Mommersteeg *et al.* 2007; Ye *et al.* 2015), we reasoned that genes with expression that correlated with *Tnni3* expression (trait ID 1422536_at, mean expression 11.552) in the whole-lung data set would also be expressed in the pulmonary myocardium. We performed sample correlation in the BXD data set and returned the top 100 significantly correlated transcripts (Supplemental Material, Table S1 in File S2; *P <* 0.0005). This set of 100 transcripts was enriched for genes known to be associated with cardiac muscle function, including structural proteins (actin and myosin), ion transporters (ryanodine receptors, calcium, and potassium transporters), and transcription factors (*Hand2* and *Nkx2-5*). To define genes within this list that were likely to act in a concerted manner in the pulmonary myocardium, we generated a network graph and found 24 transcripts that were significantly associated at a Pearson correlation >0.8. These 24 transcripts were subsequently regraphed with lower stringency to visualize potential relationships between them (Figure 1A). Three transcripts (*Tnnt2*, *Pln*, and *Kcnj3*) were detected by two separate probes and thus the 24 transcripts corresponded to only 21 unique genes. All 21 genes have previously been reported to be expressed in cardiac muscle (Chopra and Knollmann 2013; Ding *et al.* 2010; Fukuda and Mikoshiba 2000; Greulich *et al.* 2011; Guo *et al.* 2013; Hennessey *et al.* 2013; Holmegard *et al.* 2010; Huh *et al.* 2012; Marx *et al.* 2000; Reiser *et al.* 2001; Sequeira *et al.* 2015; Shaikh *et al.* 2016; Stobdan *et al.* 2015; Takeda *et al.* 2003; Warren *et al.* 2012; Yamashita *et al.* 2003; Zhou and Wu 2014) (Figure 1B and Table 1); all were highly significantly correlated with *Tnni3* (*P <* 3.02*e*^−8^). These genes can be loosely clustered into functional categories including structural sarcomeric proteins, regulation of sarcomere assembly, ion transport, transcriptional and post-transcriptional regulation, and hormone signaling (Table 1).

**Table 1:** Genes coexpressed with *Tnni3* in the BXD whole-lung transcriptome have known roles in the function and regulation of the heart

Functional Category	Gene	Name	Function
Sarcomeric structural protein	*Tnni3*	Cardiac troponin I	The troponin complex couples calcium availability with muscle contraction (Takeda *et al.* 2003)
	*Tnnt2*	Cardiac troponin T2	
	*Myh6*	Cardiac myosin heavy chain 6 (α-MHC)	Two myosin heavy chains, two essential myosin light chains and two regulatory myosin light chains interact to form the thick filament of the sarcomere (Reiser *et al.* 2001; Yamashita *et al.* 2003)
	*Myh7*	Cardiac myosin heavy chain 7 (β-MHC)	
	*Myl4*	Atrial essential myosin light chain (ALC1)	
	*Myl7*	Atrial regulatory myosin light chain (MYLC2a)	
	*Mybpc3*	Cardiac myosin binding protein C	Modulates the interaction between actin and myosin to regulate power output (Sequeira *et al.* 2015)
Regulation of sarcomere assembly	*Mylk3*	Myosin light chain kinase 3	Phosphorylates MYLC2a to facilitate actin–myosin interaction and muscle contraction (Ding *et al.* 2010; Warren *et al.* 2012)
Ion transport	*Kcnj3*	Potassium inwardly rectifying channel, subfamily J, member 3	Subunit of the muscarinic potassium channel (KCHa), important in regulation of heart rate (Holmegard *et al.* 2010)
	*Ryr2*	Cardiac ryanodine receptor 2	Mediator of SR calcium storage and release (Marx *et al.* 2000)
	*Atp2a2*	Cardiac muscle/slow twitch Ca++ transporting ATPase (SERCA2a)	Transports calcium from the cytosol to the SR lumen during muscle relaxation (Shaikh *et al.* 2016)
	*Pln*	Phospholamban	Negative regulator of SERCA2a (Shaikh *et al.* 2016)
	*Sln*	Sarcolipin	
	*Trdn*	Triadin	Forms a complex with Ryr2 to coordinate release of calcium from the SR (Chopra and Knollmann 2013)
	*Fgf12*	Fibroblast growth factor 12	Modulation of sodium and calcium channel function (Hennessey *et al.* 2013)
Transcriptional and post-transcriptional regulation	*Tbx20*	T-box 20	Activates chamber myocardial gene expression in the early heart tube (Greulich *et al.* 2011)
	*Rbm20*	RNA binding motif protein 20	Regulates alternative splicing of titin and other cardiac genes (Guo *et al.* 2013)
Hormone signaling	*Corin*	Heart-specific serine proteinase	Serine protease which activates atrial natriuretic peptide (Zhou and Wu 2014)
	*Fndc5*	Fibronectin type III domain-containing protein 5	Is cleaved to become the secreted hormone irisin (Huh *et al.* 2012)
Unknown	*Doc2g*	Double C2 gamma	Undetermined; possibly involved in calcium-dependent phospholipid binding activity (Fukuda and Mikoshiba 2000)
	*Mybphl*	Myosin binding protein H-like	Undetermined; possibly regulates cardiac function during hypoxia (Stobdan *et al.* 2015)

SR, sarcoplasmic reticulum.

To ensure that the muscle-related transcripts that we had identified were indeed associated with the pulmonary myocardium and not the smooth muscle present in small arteries and surrounding the bronchus, we performed correlation analysis using *Acta2*. The top 100 transcripts correlated with *Acta2* were enriched for smooth muscle-associated genes [including gamma-actin 2 (*Actg2*), leiomodin (*Lmod2*), transgelin (*Tagln*), and calponin 1 (*Cnn1*)], and did not significantly overlap with *Tnni3*-correlated transcripts (Table S2 in File S2).

In sum, the identity of the *Tnni3*-correlated genes themselves, and their distinct expression compared to smooth muscle-associated genes, strongly supports that we have identified a network of genes expressed in the pulmonary myocardium.

### The pulmonary myocardium gene network is coregulated from loci on Chr1 and Chr2

**Figure 2:**
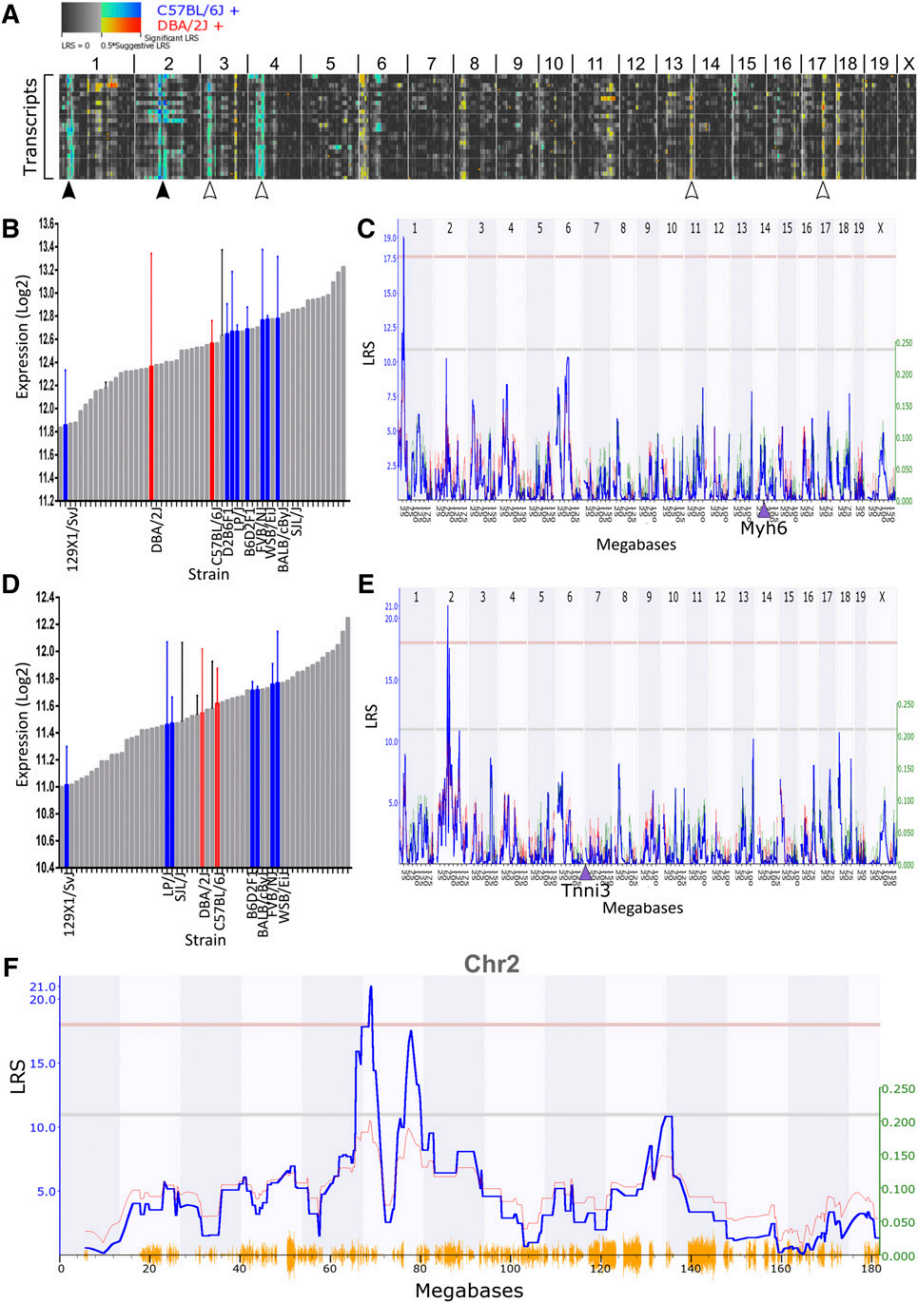
Genes within the pulmonary myocardium gene network are coregulated from loci on Chr1 and Chr2. (A) Heat-map analysis of eQTL for all 24 transcripts indicates regions of shared covariance on Chr1 and Chr2 (black arrowhead) with possible minor regions on Chr3, Chr4, Chr13, and Chr17 (white arrowhead). (B) Expression of *Myh6* varied by 2.62-fold between BXD strains. Founder strains are shown in red and non-BXD strains are shown in blue. (C) *Myh6* had a significant *trans* eQTL (LRS >17.5, red line) which mapped to Chr1: 33 ± 1 Mb. The genomic position of *Myh6* is indicated by a ▴. (D) Expression of *Tnni3* varied by 2.35-fold between BXD strains. (E) *Tnni3* had a significant *trans* eQTL on Chr2 spanning 68.8 ± 0.4 Mb. (F) *Tnni3* also had a second *trans* eQTL peak that was a suggestive eQTL (LRS >10.5, gray line) mapping to Chr2: 77.5 ± 1 Mb.

We next investigated whether transcripts within the pulmonary myocardium gene network were coregulated. Pair-wise comparisons were performed using a matrix function. All probe pairs within this set of 24 transcripts were positively correlated (Figure S1 in File S1). All pairs had a Pearson correlation >0.5 with the exception of *Myl4* and *Doc2G* (0.486), and *Myl4* and *Rbm20* (0.495). Further, heat-map analysis of eQTL for all 24 transcripts highlighted prominent regions of shared covariance on Chr1 and 2 with possible minor regions on Chr3, Chr4, Chr13, and Chr17 (Figure 2A). This suggested that expression levels of multiple transcripts in this gene network were coordinately regulated by a shared set of *cis* and *trans* eQTL.

To identify the genomic intervals harboring genes that mediated variable expression, we performed genome-wide interval mapping and marker regression analyses for each transcript. The expression level of all transcripts varied by greater than twofold between BXD strains (Table 2). All transcripts except one (*Sln*, sarcolipin) had either a suggestive (LRS >10.4) or significant (LRS >17) eQTL that mapped to the hotspot regions on Chr1 and Chr2 identified in the cluster map (Table 2). One transcript, *Myh6* (myosin heavy chain 6, Chr14), varied by 2.62-fold between the highest and lowest expressing BXD strains (Figure 2B), such that a significant *trans* eQTL could be mapped to Chr1: 33 ± 1 Mb (Figure 2C). Of the remaining transcripts, 11 also had a suggestive eQTL overlapping the Chr1: 33 ± 1 Mb region (Table 2). Two additional transcripts had significant eQTL mapping to Chr1 (*Doc2g*, Chr19; *Trnd*, Chr10) but these did not overlap and were not replicated in more than one other transcript (Table 2). Two transcripts, *Kcnj3* and *Tnni3* (Chr2 and Chr7, respectively), had overlapping significant eQTL on Chr2. For example, *Tnni3* expression varied by 2.35-fold between strains (Figure 2D) and a *trans* eQTL was mapped to Chr2: 68.8 ± 0.4 Mb (Figure 2E). Of the remaining transcripts, 12 also had a suggestive eQTL overlapping the Chr2: 68.8 ± 0.4 Mb region (Table 2).

**Table 2:** All transcripts except sarcolipin were regulated from an eQTL that mapped to Chr1 and/or Chr2

Gene Name	Location	Range (-fold)	eQTL			
			Chromosome	Location*^a^*	Genes	LRS
*Tnni3*	Chr7: 4.469	2.38	Chr2	68.4–69.2	9	>17.78
			Chr2	76.4–80.1	20	>11.02
*Mybphl*	Chr3: 108.178	2.90	Chr1	32.5–34.5	15	>10.93
			Chr2	77.8–78.7	6	
			Chr2	68.4–69.5	11	
			Chr4	49.6–54.9	27	
			Chr17	62.9–64	5	
*Myl7*	Chr11: 5.796	2.65	Chr2	68.5–69.5	13	>10.92
			Chr17	62.9–64	5	
			Chr18	9.6–11	14	
*Tnnt2*	Chr1: 137.745	2.51	Chr2	68.9–69.2	6	>10.87
			Chr13	116–116.35	1	
*Pln*	Chr10: 53.065	4.15	Chr2	65.5–68.9	19	>10.77
			Chr2	77.5–94	362	
			Chr2	113.4–113.7	8	
			Chr2	133.2–135.9	10	
*Myh6*	Chr14: 55.560	2.62	Chr1	32.2–34	9	>17.62
			Chr1	23–25.1	17	>10.88
*Fndc5*	Chr4: 128.821	2.26	Chr2	65.5–69.7	32	>10.77
			Chr2	79–80	7	
*Atp2a2*	Chr5: 122.903	2.03	Chr1	32.2–34.5	15	>10.89
			Chr2	77.5–78.5	2	
			Chr4	29–32	7	
*Mylk3* (*D830007F02Rik*)	Chr8: 87.848	2.95	Chr1	32.5–34	9	>10.77
			Chr2	68.6–69.4	8	
*Ryr2*	Chr13: 11.645	3.09	Chr1	32.5–35	24	>10.84
			Chr4	31–32.5	4	
*Kcnj3*	Chr2: 55.450	5.40	Chr2	65.7–70.8	32	>16.51
			Chr3	35.8–37	9	>10.48
			Chr6	14–17	6	
*Mybpc3*	Chr2: 90.975	4.16	Chr2	76.5–93.8	365	>16.98
			Chr2	65.5–70.5	36	>10.6
*Myh7*	Chr14: 55.590	2.89	Chr1	32–34.5	15	>10.99
			Chr6	67.5–76	103	
			Chr17	63–64	5	
*Sln*	Chr9: 53.698	2.37	Chr17	57–66	45	>10.92
*Doc2g*	Chr19: 4.006	2.32	Chr1	23–25.5	17	>17.35
			Chr2	68.6–70.3	20	>10.82
			Chr6	13–15	11	
*Tnnt2_2*	Chr1: 137.747	2.33	Chr1	32.2–34.5	15	>10.89
*Rbm20* (*1110018J23Rik*)	Chr19: 53.941	2.60	Chr2	76–94	365	>10.84
			Chr2	68.9–69.3	8	
			Chr4	49.5–54.9	32	
			Chr6	56.5	2	
*Pln_2*	Chr10: 53.063	4.17	Chr1	32.2–35	24	>10.75
			Chr2	77.5–78.5	2	
			Chr2	133.2–135.9	9	
*Trdn*	Chr10: 32.919	2.44	Chr1	154.7–155.8	13	>17.4
			Chr1	145.4–146	9	>10.7
			Chr1	160.17–160.29	2	
			Chr2	65.5–69.8	36	
			Chr3	28–36	60	
*Corin*	Chr5: 72.691	3.61	Chr1	32.5–35	24	>10.73
			Chr4	31.5–36	38	
*Myl4*	Chr11: 104.445	3.56	Chr11	100.5–107	171	>10.89
			Chr1	32–34.5	13	
			Chr2	69–69.5	10	
*Kcnj3_2*	Chr2: 55.449	2.84	Chr2	68.6–69.8	20	>10.83
			Chr2	88–92	135	
*Fgf12*	Chr16: 28.189	2.56	Chr1	32.5 –34.5	15	>10.99
			Chr6	22–24.5	18	
*Tbx20*	Chr9: 24.525	3.17	Chr1	32.5–34.5	15	>10.94
			Chr3	28–36.5	67	

a Location (megabase) was defined as the genomic coordinates at which the eQTL peak crosses the suggested or significant LRS threshold.

A second eQTL peak on Chr2: 77.5 ± 1 Mb also reached significance for one gene (*Mybpc3*, Chr2) and was a suggestive eQTL for seven additional transcripts (Table 2). Using the *Tnni3* eQTL plot as an example, this Chr2: 77.5 ± 1 Mb region had a suggestive LRS (Figure 2F). Since three significant eQTL intervals (Chr1: 33 ± 1 Mb, Chr2: 68.8 ± 0.4 Mb, and Chr2: 77.5 ± 1 Mb) were mapped with suggestive LRS in multiple pulmonary myocardium transcripts, the genes within these regions were interrogated to identify putative upstream regulatory genes.

### Rab23, Zfp451, Nostrin, Lrp2, and Ttn are candidate regulators of the pulmonary myocardium gene network

The genes within the peak linkage regions were subsequently analyzed for known biological functions (GeneCards) (Safran *et al.* 2010) and expression in lung tissue [Fantom5 (Forrest *et al.* 2014; Severin *et al.* 2014); Table 3]. The Chr1: 33 ± 1 Mb interval contained nine genes, with five of these (*Prim2*, *Rab23*, *Bag2*, *Zfp451*, and *Dst*) expressed in adult lung (Table 3). Both *Rab23* and *Zfp451* have known functions that could implicate them in regulating gene expression of the pulmonary myocardium network. RAB23, a GTPase of the RAB family involved in vesicular trafficking, also exerts downstream transcriptional effects by antagonizing the action of the transcription factor GLI1 (Sun *et al.* 2012). However, the function, localization, and mechanisms of action of RAB23 are still largely undefined. On the other hand, ZFP451 (zinc finger protein 451) is a promyelocytic leukemia nuclear body-associated transcriptional coregulator, and modulates transcription by regulating protein subnuclear localization (Karvonen *et al.* 2008).

**Table 3:** Identification, function, and transcript expression of genes within an eQTL on Chr 1 and 2

Linkage Peak	Gene	SNP Count	Function (GeneCards)	Fantom5 (TPM)*^a^*
Chr1: 32 Mb	*Khdrbs2*	630	RNA-binding protein that plays a role in the regulation of alternative splicing	<1
	*EG408191*	10	Predicted gene	None
	*Prim2*	16	DNA primase, p58 subunit; an enzyme that plays a key role in the replication of DNA	6
	*1700001G17Rik*	0	Uncharacterized protein, no biological data	<1
	*Rab23*	1	This gene encodes a small GTPase of the Ras superfamily; may play a role in central nervous system development by antagonizing SHH signaling	4.06
	*Bag2*	2	Competes with Hip for binding to the Hsc70/Hsp70 ATPase domain and promotes substrate release	3.51
	*Zfp451*	16	Involved in transcriptional regulation; coactivator for steroid receptors	9.79
	*Bend6*	31	Among its related pathways are Notch signaling pathways	<1
	*Dst*	340	This gene encodes a member of the plakin protein family of adhesion junction plaque proteins	20.14
Chr2: 69 Mb	*4932414N04Rik*	117	Uncharacterized protein, no biological data	None
	*Lass6*	33	Related pathways are sphingolipid metabolism	47.11
	*2010109K09Rik*	0	Uncharacterized protein, no biological data	None
	*Nostrin*	10	A potent mediator in biologic processes such as neurotransmission, inflammatory response, and vascular homeostasis	106.79
	*Spc25*	2	Involved in kinetochore–microtubule interaction and spindle checkpoint	None
	*G6pc2*	0	Enzyme belonging to the glucose-6-phosphatase catalytic subunit family	None
	*Abcb11*	41	Member of the superfamily of ATP-binding cassette (ABC) transporters	None
	*Dhrs9*	54	This protein demonstrates oxidoreductase activity and may additionally function as a transcriptional repressor in the nucleus	None
	*Lrp2*	199	LRP2 protein is critical for the reuptake of numerous ligands, including lipoproteins, sterols, vitamin-binding proteins, and hormones	36.58
Chr2: 78 Mb	*Ttn*	7	This gene encodes titin, a large abundant protein of striated muscle; titin also contains binding sites for muscle-associated proteins	24.76
	*Ccdc141*	2	Role in radial migration and centrosomal function	43.05
	*Sestd1*	4	Docking protein directing membrane turnover	<1
	*Zfp385b*	2	Nucleic acid binding and p53 binding	<1
	*Cwc22*	1	Required for pre-mRNA splicing and for exon–junction complex assembly	<1
	*4930440I19Rik*	15	Uncharacterized protein, no biological data	None

TPM, tags per million.

The Chr2: 68.8 ± 0.4 Mb interval contained nine genes, three of which were expressed at appreciable levels in adult lung (*Lass6*, *Nostrin*, and *Lrp2*) but only two had known roles in gene regulation (*Nostrin*) or cell signaling (*Lrp2*) (Table 3). Nostrin has been shown to be required for vascular development and angiogenesis by acting as a molecular conduit for fibroblast growth factor receptor 1 (FGFR1)-mediated induction of Ras-related C3 botulinum toxin substrate 1 (RAC1) activity (Kovacevic *et al.* 2012). Interestingly, Nostrin has been shown in both mice (Kim *et al.* 2005) and humans (Wiesenthal *et al.* 2009) to bind to and negatively regulate transcription of its own promoter via a centrally located bZIP DNA binding motif (Bae *et al.* 2014).

However, transcriptional targets apart from *Nostrin* itself have not yet been found. LRP2 (low density lipoprotein receptor-related protein 2) is a critical component of the sonic hedgehog (SHH) signaling pathway (Christ *et al.* 2012). There is some evidence that LRP2 can shed its intracellular domain, which then shuttles to the nucleus where it may interact with as-yet-unidentified transcription factors (Li *et al.* 2008). However, a role for LRP2 has not yet been identified in either heart or lung. The Chr2: 77.5 ± 1 Mb region contained six genes, only two of which were expressed in adult lung (*Ttn* and *Ccdc141*; Table 3). Of these, titin (*Ttn*) was a likely candidate modifier based on its role as a key integrator of myocyte signaling pathways (Linke 2008; Miller *et al.* 2004). Therefore, *Rab23*, *Zfp451*, *Nostrin*, *Lrp2*, and *Ttn* were analyzed further.

### The transcription factors NKX2-5, SRF, MEF2A, TEAD1, and NR2F1 potentially cooperate to specify and/or maintain pulmonary myocardium identity

Since NKX2-5 has previously been shown to be important for the maintenance of pulmonary myocardium cell identity, we investigated the possibility that genes within the pulmonary myocardium, and the candidate upstream regulators, might be direct targets of NKX2-5. To this end, we interrogated previously published NKX2-5 ChIP-seq data derived from two sources. He *et al.* (2011) used a doxycycline-inducible dual adenovirus system to express biotinylated NKX2-5 in the HL1 cardiomyocyte cell line, while Dupays *et al.* (2015) examined endogenous NKX2-5 binding in mouse hearts at embryonic day 11.5.

Of the 21 genes in our gene network, NKX2-5 binding sites were detected within 10 kb of seven genes (*Rbm20*, *Corin*, *Myl7*, *Tnnt2*, *Ryr2*, *Fgf12*, and *Myh7*) in the He *et al.* data set, two genes (*Mybpc3* and *Trdn*) in the Dupays *et al.* data set, and three genes (*Tbx20*, *Mybphl*, and *Atp2a2*) in both data sets (Dupays *et al.* 2015; He *et al.* 2011) (Table 4). Collectively, this indicates that at least 50% of the genes in the pulmonary myocardium gene network are bound by NKX2-5 in heart tissue at some point during development. We also examined NKX2-5 enrichment at our candidate pulmonary myocardium control loci on Chr1 and Chr2 and found NKX2-5 enrichment at *Rab23*, *Zfp451*, and *Nostrin* in the He *et al.* data set. Although tissue-specific regulatory mechanisms may differ between the heart and the pulmonary myocardium, this is strong evidence to support the theory that NKX2-5 could directly regulate expression of the pulmonary myocardium gene network.

**Table 4:** ChIP-seq enrichment of Nkx2-5, Srf, and Mef2a in genomic regions proximal to genes within the pulmonary myocardium gene network and candidate upstream regulators

Gene	Correlated with NKX2-5 (*P* < 0.005)	Dupay *et al.* (2015)			He *et al.* (2011)		
		NKX2-5	SRF	MEF2A	NKX2-5	SRF	MEF2A
*Tnni3*	Yes, 0.613	No	No	No	No	No	No
*Mybphl*	Yes, 0.563	Yes	No	Yes	Yes	Yes	No
*Myl7*	Yes, 0.454	No	No	No	Yes	Yes	No
*Tnnt2*	Yes, 0.426	No	Yes	Yes	Yes	Yes	Yes
*Pln*	Yes, 0.539	No	No	Yes	No	No	No
*Myh6*	Yes, 0.454	No	Yes	Yes	No	No	No
*Fndc5*	Yes, 0.427	No	No	No	No	Yes	No
*Atp2a2*	No	Yes	Yes	Yes	Yes	Yes	Yes
*Mylk3* (*D830007F02Rik*)	Yes, 0.501	No	No	No	No	No	No
*Ryr2*	Yes, 0.394	No	No	No	Yes	Yes	No
*Kcnj3*	Yes, 0.397	No	No	No	No	No	No
*Mybpc3*	Yes, 0.597	Yes	No	Yes	No	Yes	No
*Myh7*	Yes, 0.421	No	No	No	Yes	Yes	Yes
*Sln*	Yes, 0.459	No	No	No	No	No	No
*Doc2g*	Yes, 0.631	No	No	No	No	No	No
*Rbm20* (*1110018J23Rik*)	Yes, 0.585	No	No	No	Yes	Yes	No
*Trdn*	Yes, 0.466	Yes	Yes	Yes	No	No	No
*Corin*	No	No	No	No	Yes	Yes	Yes
*Myl4*	Yes, 0.510	No	No	No	No	Yes	No
*Fgf12*	Yes, 0.404	No	No	No	Yes	Yes	No
*Tbx20*	No	Yes	Yes	Yes	Yes	Yes	No
*Nostrin*	No	No	No	No	Yes	No	No
*Rab23*	No	No	No	No	Yes	Yes	No
*Zfp451*	No	No	No	No	Yes	Yes	No
*Lrp2*	No	No	No	No	No	No	No
*Ttn*	No	No	Yes	Yes	No	Yes	Yes

To further define transcriptional programs that might specify pulmonary myocardium identity, we performed transcription factor binding site enrichment analysis. We used the oPOSSUM-3 Web-based tool (Kwon *et al.* 2012) to identify overrepresented, conserved transcription factor binding sites in our set of 21 pulmonary myocardium genes and five candidate modifier genes. The pulmonary myocardium gene network was enriched in binding site sequences for SRF (serum response factor; 10 genes), TEAD1 (TEA Domain Family Member 1; 14 genes), NR2F1 (Nuclear Receptor Subfamily 2, Group F, Member 1; 12 genes), and MEF2A (Myocyte Enhancer Factor 2A; 18 genes). Interestingly, while SRF, MEF2A, and TEAD1 were reported to have positive roles in regulating cardiac development (He *et al.* 2011), NR2F1 has been shown to antagonize the activity of NKX2-5 (Guo *et al.* 2001). Since SRF and MEF2A were both examined by ChIP-seq in the He *et al.* (2011) and Dupays *et al.* (2015) data sets, we also investigated their enrichment in the genomic regions proximal to our genes of interest. Either one or both factors were enriched in 17 out of 21 genes (Table 4). Therefore, these transcription factors could form part of a regulatory network that directs or maintains the identity of the pulmonary myocardium.

We subsequently investigated the expression of *Nkx2-5*, *Srf*, *Mef2a*, *Tead1*, and *Nr2f1* in the lung using the Fantom5 mouse promoterome database (data not shown). At the transcript level, the expression of all factors except *Nkx2-5* could be detected in the lung (lung, adult: CNhs10474 ctss). The absence of *Nkx2-5* expression in adult lung is an apparent paradox since this factor has previously been shown to be required for pulmonary myocardium identity (Millino *et al.* 2000; Mommersteeg *et al.* 2007). However, whether the lung sections sampled in the Fantom5 project actually contain pulmonary myocardium cannot be determined. In contrast, *Nkx2-5* low-level expression in the BXD lung data set could be detected with a mean of 7.798 log_2_ units [background is generally considered to be <7 log_2_ units (Geisert *et al.* 2009)]. Further, *Nkx2-5* expression in the lung data set was significantly positively correlated with almost all the members of the pulmonary myocardium gene network (Table 4).

### The pulmonary myocardium gene network and coexpressed genes are associated with cardiac phenotypes overlapping those of the stressed heart

A limitation to single trait/transcript analysis is that it does not allow for combinatorial interactions with additional genetic loci, because each trait is individually regressed against every marker, *i.e.*, only one locus is considered per trait (Michaelson *et al.* 2009). Therefore, all 24 pulmonary myocardium gene network transcripts were merged into a single trait using PCA. A network of 100 probes that correlated (*r >* 0.553, *P <* 2.40*e*^−05^) with the synthetic PCA trait was identified. Of the 100 probes, 76 had unique Entrez Gene IDs (Table S3 in File S2). These genes were considered to be part of a pulmonary myocardium gene coexpression network. This extended gene network contained a number of transcription factors (*Tbx5*, *Hand2*, *Fhl2*, and *Gata4*) and ion/calcium handling genes (*Ank2*, *Cacna2d2*, *Scn5a*, *Kcnd2*, *Kcnj5*, *Myoz2*, *Casq2*, *Csrp3*, and *Gja3*) of interest in the context of atrial fibrillation.

We used this pulmonary myocardium gene coexpression network to investigate enriched GO terms, signaling pathways, and human phenotypes related to the coexpression of these genes in the lung using WebGestalt (Wang *et al.* 2013). The pulmonary myocardium gene coexpression network was significantly enriched with GO terms involving biological processes related to both striated and cardiac muscle function and development (Table 5). Enriched molecular functions included cytoskeletal protein binding and cation transmembrane transporter activity (Table 5), and enriched cellular components included contractile fiber and sarcomeric genes (Table 5). Collectively, these data support our approach for identifying genes that are likely to be expressed in the pulmonary myocardium, as our coexpressed genes are indeed enriched for cardiac muscle function.

**Table 5:** Enriched GO terms and signaling pathways related to the coexpression of pulmonary myocardium-associated genes

Category	Name	Genes	*P* Value*^a^*
Biological process	Muscle system process	21	3.04*e*^−21^
	Muscle contraction	18	3.70*e*^−18^
	Blood circulation	20	1.63*e*^−16^
	Circulatory system process	20	1.63*e*^−16^
	Heart contraction	15	9.39*e*^−16^
	Heart process	15	9.39*e*^−16^
	System process	31	9.18*e*^−15^
	Striated muscle contraction	12	3.35*e*^−14^
	Cardiac muscle tissue development	14	4.36*e*^−14^
	Striated muscle tissue development	17	4.23*e*^−13^
Molecular function	Cytoskeletal protein binding	20	4.63*e*^−11^
	Titin binding	4	3.04*e*^−06^
	Protein binding	47	7.20*e*^−06^
	Inorganic cation transmembrane transporter activity	11	2.66*e*^−05^
	Cation transmembrane transporter activity	11	0.0001
	Monovalent inorganic cation transmembrane transporter activity	8	0.0002
	Metal ion transmembrane transporter activity	9	0.0002
	Actin binding	9	0.0002
	Transmembrane transporter activity	13	0.0005
	Ion channel binding	4	0.0006
Cellular component	Sarcomere	20	3.94*e*^−24^
	Contractile fiber	21	6.59*e*^−24^
	Contractile fiber part	20	9.05*e*^−24^
	Myofibril	20	2.56*e*^−23^
	I-band	12	1.36*e*^−13^
	A-band	8	6.44*e*^−13^
	Z-disc	11	7.63*e*^−13^
	Sarcoplasmic reticulum	9	1.12*e*^−11^
	Sarcoplasm	9	3.02*e*^−11^
	M-band	5	1.46*e*^−08^
KEGG pathway	Dilated cardiomyopathy	11	5.71*e*^−18^
	Cardiac muscle contraction	10	1.12*e*^−16^
	Hypertrophic cardiomyopathy	10	1.12*e*^−16^
	Calcium signaling pathway	7	1.90*e*^−08^
	Arrhythmogenic right ventricular cardiomyopathy	4	1.06*e*^−05^
	Tight junction	4	0.0001
	Focal adhesion	4	0.0003
	Regulation of actin cytoskeleton	4	0.0005
WikiPathway	Striated muscle contraction	11	7.67*e*^−22^
	Calcium regulation in the cardiac cell	7	7.75*e*^−09^
	Heart development	4	4.95*e*^−06^
	Myometrial relaxation and contraction pathways	5	6.19*e*^−06^
	miR-1 in cardiac development	2	1.12*e*^−05^
	MicroRNAs in cardiomyocyte hypertrophy	4	1.94*e*^−05^
	G protein signaling pathways	3	0.0005

a Adjusted for multiple tests.

**Figure 3:**
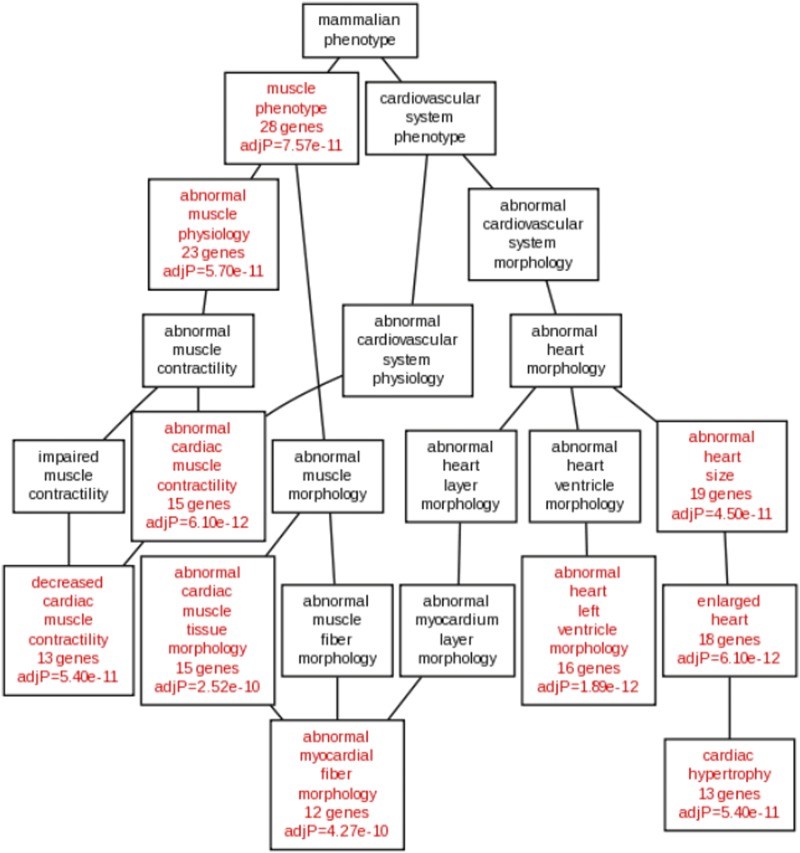
Human phenotypes associated with the pulmonary myocardium coexpression gene network (PM-CGN) are associated with abnormal cardiac function. Phenotypes associated with the PM-CGN are related to muscle and cardiovascular system dysfunction. Pathways enriched with genes from the PM-CGN are shown in red.

Interrogation of related signaling pathways with the KEGG pathway tool revealed enrichment for “dilated cardiomyopathy,” “cardiac muscle contraction,” “hypertrophic cardiomyopathy,” and “calcium signaling pathway” genes (Table 5). Similarly, analyses with the WikiPathway tool demonstrated enrichment for “striated muscle contraction” and “calcium regulation in the cardiac cell” (Table 5). In agreement with the GO and pathway results, the pulmonary myocardium gene coexpression network was enriched with higher order muscle phenotypes such as “abnormal cardiac muscle contractility,” “abnormal cardiac tissue morphology,” and “abnormal heart size” (Figure 3). Enrichment for pathways related to both abnormal contractility and calcium signaling/regulation led us to investigate the ion channels and regulatory proteins expressed in the extended gene network.

### The expression of calcium handling networks in the pulmonary myocardium are dysregulated in atrial fibrillation

We chose to investigate the potential for genes involved in ion transport and calcium handling to be dysregulated in atrial fibrillation. We selected 16 genes which are known to be involved in regulation of ion transport, almost all of which are also known susceptibility genes for arrhythmia (Table 6) (Altmann *et al.* 2015; Bos *et al.* 2009; Chi *et al.* 2010; Frank-Hansen *et al.* 2005; Freyermuth *et al.* 2016; Holmegard *et al.* 2010; Kokunai *et al.* 2014; Liu *et al.* 2015; Mohler *et al.* 2003; Musa *et al.* 2015; Osio *et al.* 2007; Roux-buisson *et al.* 2012; Shan *et al.* 2012; Shanmugam *et al.* 2011; Song *et al.* 2007; Wang *et al.* 1995), and analyzed their expression in a publicly available mRNA microarray data set generated by Deshmukh *et al.* (2015). This study used RNA extracted from left atrial appendage tissue obtained from cardiac surgery patients who were divided into three categories: those with atrial fibrillation who either were or were not in sinus rhythm at the time tissue samples were collected, and those with no history of atrial fibrillation. The patient cohorts represent susceptibility to atrial fibrillation and persistent atrial fibrillation, respectively, when compared to control samples with no history of atrial fibrillation (Deshmukh *et al.* 2015). We compared our genes of interest (Table 6) to expression changes in this data set to determine if genes in our network are likely to be dysregulated in atrial fibrillation. Of these 16 genes, four were significantly downregulated (*Cacna2d2*, 0.5-fold; *Kcnj5*, 0.7-fold; *Myoz2*, 0.7-fold; and *Sln*, 0.7-fold) and three were significantly upregulated (*Atp2a2*, 1.6-fold; *Csrp3*, 1.8-fold; and *Gja3*, 1.6-fold) in the persistent atrial fibrillation cohort compared to either the susceptibility or the control cohorts (Table 6).

**Table 6:** The pulmonary myocardium gene coexpression network contains genes involved in ion transport that are associated with arrhythmia and dysregulated in atrial fibrillation

Genes Involved in Ion Handling				Deshmukh *et al.* (2015)			
Gene	Name	Function	Association with Arrhythmia	*P* Value	FDR < 0.05 *P* Value	Fold-change	Cohort
*Ank2*	Ankyrin B (neuronal)	Required for targeting and stability of Na/Ca exchanger 1 in cardiomyocytes	Type 4 long-QT cardiac arrhythmia (Mohler *et al.* 2003)	No change			
*Cacna2d2*	Calcium channel, voltage-dependent, α-2/δ subunit 2	This gene encodes the α-2/δ subunit of the voltage-dependent calcium channel complex.	No evidence	3.5*e*^−08^	1.338*e*^−06^	0.58825	AF/AF *vs.* AF/SR
*Scn5a*	Sodium channel, voltage-gated, type V α subunit	This protein mediates the voltage-dependent sodium ion permeability of excitable membranes; responsible for the initial upstroke of the action potential in an electrocardiogram	Long QT syndrome type 3 and myotonic dystrophy (Freyermuth *et al.* 2016; Wang *et al.* 1995; Musa *et al.* 2015)	No change			
*Ryr2*	Ryanodine receptor 2 (cardiac)	Mediator of SR calcium storage and release	Atrial fibrillation (Shan *et al.* 2012)	No change			
*Kcnd2*	Voltage-gated potassium channel subunit Kv4.2	This gene encodes a voltage-activated A-type potassium ion channel prominent in the repolarization phase of the action potential.	No evidence	No change			
*Kcnj3*	Potassium inwardly rectifying channel, subfamily J, member 3	Subunit of the muscarinic potassium channel (KCHa), important in regulation of heart rate	No evidence (Holmegard *et al.* 2010)	No change			
*Kcnj5*	Potassium inwardly rectifying channel, subfamily J, member 5	The encoded protein may associate with two other G-protein-activated potassium channels to form a multimeric pore-forming complex.	Andersen–Tawil syndrome (Kokunai *et al.* 2014)	0.00112	0.0091709	0.71886	AF/AF *vs.* AF/SR
*Atp2a2*	Cardiac muscle/slow twitch Ca++ transporting ATPase	Transports calcium from the cytosol to the SR lumen during muscle relaxation	No evidence	4.5*e*^−07^	1.242*e*^−05^	1.62812	AF/AF *vs.* AF/SR
*Trdn*	Triadin	Forms a complex with Ryr2 to coordinate release of calcium from the SR	Ventricular tachycardia, long QT syndrome (Roux-buisson *et al.* 2012; Altman *et al.* 2015)	No change			
*Myoz2*	Myozenin 2	The protein encoded by this gene binds to calcineurin, a phosphatase involved in calcium-dependent signal transduction in diverse cell types.	Hypertrophic cardiomyopathy with arrhythmia (Osio *et al.* 2007)	0.00014	0.0017485	0.73941	AF/AF *vs.* AF/SR
*Casq2*	Calsequestrin 2 (cardiac muscle)	The protein is a calcium binding protein that stores calcium for muscle function.	Catecholaminergic polymorphic ventricular tachycardia (Song *et al.* 2007)	No change			
*Csrp3*	Cysteine and glycine-rich protein 3 (cardiac LIM protein)	Plays a crucial and specific role in the organization of cytosolic structures in cardiomyocytes and is essential for calcineurin anchorage to the Z-line.	Hypertrophic cardiomyopathy (Bos *et al.* 2009)	3.3*e*^−11^	2.385*e*^−09^	1.82455	AF/AF *vs.* AF/SR
*Pln*	Phospholamban	Negative regulator of SERCA2a	Ventricular arrhythmia (Liu *et al.* 2015)	No change			
*Sln*	Sarcolipin	Negative regulator of SERCA2a	Atrial fibrillation (Shanmugam *et al.* 2011)	0.00511	0.0303076	0.76185	AF/AF *vs.* AF/SR
*Fgf12*	Fibroblast growth factor 12	Modulation of sodium and calcium channel function	Atrial fibrillation (Musa *et al.* 2015)	No change			
*Gja3*	Gap junction protein, α-3, 46 kDa (Connexin 46)	The protein encoded by this gene is a connexin and is a component of lens fiber gap junctions.	Heart failure and uncoordinated ventricular contraction in zebrafish (Chi *et al.* 2010)	0.00021	0.0056234	1.68501	AF/AF *vs.* NoAF

AF/AF, indicative of persistent atrial fibrillation; AF/SR, indicative of susceptibility to atrial fibrillation; SR, sarcoplasmic reticulum; NoAF, a cohort with no history of atrial fibrillation.

We also compared our original gene network, putative upstream regulators, and transcription factors from the extended gene network with the Deshmukh *et al.* data set and found three genes were upregulated (*Myh7*, 1.8-fold; *Ttn*, 1.8-fold; and *Fhl2*, fourfold) and five genes were downregulated (*Rbm20*, 0.6-fold; *Tnnt2*, 0.6-fold; *Fndc5*, 0.7-fold; *Mybphl*, 0.5-fold; and *Tbx5*, 0.7-fold) in the persistent atrial fibrillation cohort compared to either the susceptibility or the control cohorts (Deshmukh *et al.* 2015).

The dysregulation of these genes in atrial tissue of patients with persistent atrial fibrillation, especially *Ttn* which we propose acts upstream of many of the genes in our network, highlights the need for similar gene expression studies to be performed with pulmonary myocardium tissue.

### Gene network analysis effectively identifies markers expressed in the pulmonary myocardium from whole-lung transcriptome data

To confirm that our analyses have, in fact, identified genes expressed in the pulmonary myocardium, lung tissue samples were analyzed from four BALB/c mice using immunohistochemistry to detect the cellular localization of TNNI3 and ACTC1. ACTC1 was selected as its transcript was highly expressed in the lung data set (12.422 units) and was part of our pulmonary myocardium gene coexpression network (Table S3 in File S2). Further, *Actc1* has previously been shown to be a direct target of NKX2-5 (Chen *et al.* 1996). To differentiate between the pulmonary myocardium and typical smooth muscle, we also examined the localization of ACTA2. Cardiac ventricular tissue was used as a positive control for cardiomyocyte (TNNI3 and ACTC1) and smooth muscle (ACTA2) immunostaining (Figure S2 in File S1).

**Figure 4:**
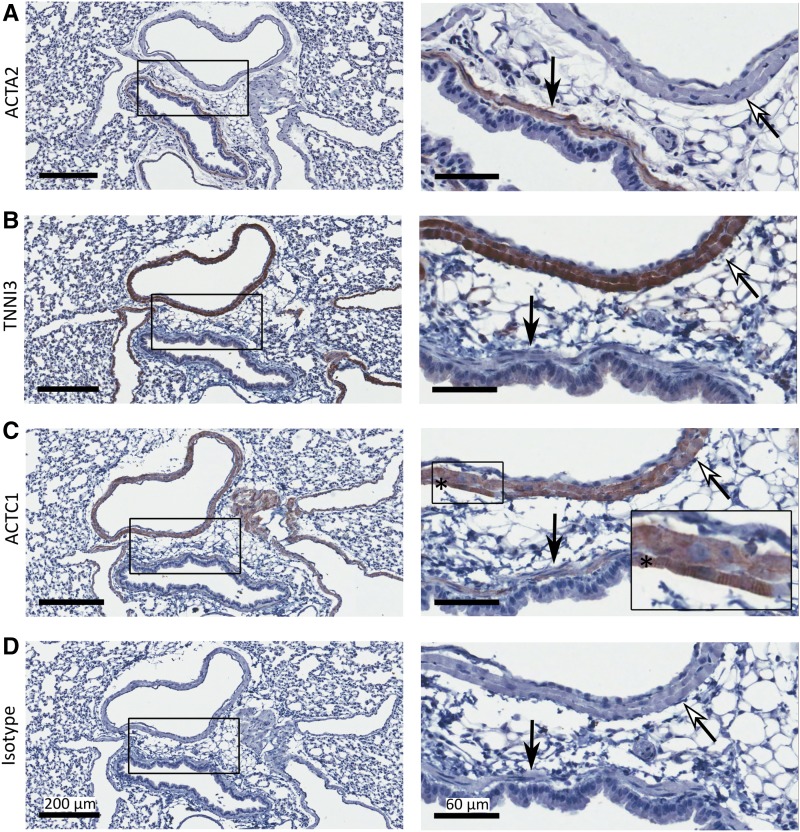
TNNI3 and ACTA2 are localized to different structures in mouse lung. Serial sections of lung tissue were immunostained for (A) ACTA2, (B) TNNI3, (C) ACTC1, and (D) IgG isotype control (Isotype). Images in right-hand panels (bar, 60 μm) show detail from black boxes in left-hand panels (bar, 200 μm). Distinct cell-type localization of TNNI3 in striated muscle (white ←) and ACTA2 in smooth muscle (black ←) are indicated. Muscle striations are shown in (C) (*).

Distinct cell-type localization of TNNI3 and ACTA2 was observed in lung samples from four BALB/c mice (Figure 4; representative image from one mouse shown). A thin muscle coat staining positively for ACTA2 was situated beneath the columnar epithelium (Figure 4A, black filled arrow) typical of bronchi and bronchioles in BALB/c mice (Rockx *et al.* 2007). Consistent with previous reports describing the pulmonary vein myocardium (Kracklauer *et al.* 2013; Mueller-Hoecker *et al.* 2008), a thick muscle coat positively staining for TNNI3 was situated beneath a monolayer of endothelium lining the vein lumen (Mueller-Hoecker *et al.* 2008) (Figure 4B, white filled arrow). This myocardial layer also stained positively for ACTC1 (Figure 4C, white filled arrow), and contained visible striations (Figure 4C, insert with *) previously reported to be evident in pulmonary vein myocardium (Kracklauer *et al.* 2013; Mueller-Hoecker *et al.* 2008). Weak staining of ACTC1 but not TNNI3 was detected in bronchial smooth muscle cells (Figure 4, B and C, black filled arrow). No positive staining was detected with an isotype control antibody (Figure 4D).

## Discussion

Numerous studies now indicate that polymorphisms in genes encoding the cardiac transcription factors NKX2-5 and PITX2C increase susceptibility to atrial fibrillation (Huang *et al.* 2013; Qiu *et al.* 2014; Wang *et al.* 2014; Xie *et al.* 2013), suggesting the potential involvement of disrupted gene networks in this disorder. Both factors are required for development of the pulmonary myocardium (Mommersteeg *et al.* 2007; Ye *et al.* 2015), which is also thought to have a pathological role in atrial fibrillation (Chen *et al.* 1999). A large body of literature has also been amassed to show that variants in genes regulating calcium handling lead to arrhythmia (Table 6). However, how the pulmonary myocardium gains pacemaker activity in atrial fibrillation patients is still unknown. Further, the underlying molecular identity of the pulmonary myocardium, including which ion channels and transcription factors are expressed in this tissue, has not been described in detail. We have used a systems-genetics approach to examine a large network of coexpressed genes simultaneously without prior knowledge of their identity, and have attempted to shed light on the relationships between transcriptional regulation, ion/calcium handling, contractile phenotypes, and the gene networks involved. By correlating variation in gene expression and known BXD genotypes in a genome-wide eQTL analysis we identified three regulatory loci associated with the expression of a cardiac gene network in the pulmonary myocardium.

The best candidate gene in the Chr2: 68.8 ± 0.4 Mb interval was *Nostrin* which is expressed in endothelial cells, heart, and highly vascularized tissues including the lung (Zimmermann *et al.* 2002), and has clear potential as a regulatory molecule. A primary function of NOSTRIN is to modulate the activity of endothelial nitric oxide synthase (eNOS) by sequestering eNOS away from the plasma membrane (Zimmermann *et al.* 2002). In turn, this prevents the calcium-mediated release of nitric oxide (NO) (Zimmermann *et al.* 2002), a potent modifier of cardiac function, including regulation of contractility (Massion *et al.* 2003; Petroff *et al.* 2001). Alternative splicing of *Nostrin* produces a truncated isoform (NOSTRIN-β) which is primarily localized to the nucleus (Wiesenthal *et al.* 2009) and represses transcription from its own promoter (Kim *et al.* 2005; Wiesenthal *et al.* 2009). Therefore NOSTRIN-β could also influence transcription of other genes within the pulmonary myocardium gene network. A model can be envisaged where NOSTRIN-β alters expression of RBM20 (the linkage peak for the *Rbm20* eQTL contains *Nostrin*), leading to altered splicing of *Nostrin* (as well as other key myocyte genes), to provide feedback from stretch-induced NO release and result in altered contractile properties. Interestingly, RBM20 has been shown to directly influence alternative splicing of *Zfp451* and *Ttn* (Guo *et al.* 2013), both of which were identified as candidate upstream regulators in the other two eQTL peaks identified in this study.

The eQTL interval located at Chr1: 33 ± 1 Mb contained two possible candidates for regulating expression of the pulmonary myocardium gene network: *Rab23* and *Zfp451*. Since a defined relationship exists between ZFP451 and cardiac function, we suggest that ZFP451 is the better candidate. ZFP451 has been shown to bind directly to SMAD3/4 which prevents recruitment of p300 to target promoters and results in the downregulation of transforming growth factor-β (TGFβ) target genes in A549 lung epithelial cells (Feng *et al.* 2014). This is relevant since transgenic mice expressing a constitutively active form of TGFβ_1_ were shown to develop atrial but not ventricular fibrosis and had increased susceptibility to atrial fibrillation (Nakajima *et al.* 2000; Verheule *et al.* 2004). Upregulation of TGFβ_1_ is also known to induce myocardial hypertrophy (Parker *et al.* 1990) and is evident in animal models of heart failure (Dobaczewski *et al.* 2011). However, therapies which block TGFβ would be expected to have adverse effects due to the pleiotropic nature of TGFβ signaling (Dobaczewski *et al.* 2011). In future studies, it will be important to determine if ZFP451 expression in the pulmonary myocardium and heart of atrial fibrillation patients is altered in parallel with increased TGFβ expression; if so, this transcription factor might be a viable therapeutic target.

A likely candidate underlying the Chr2: 77.5 ± 1 Mb eQTL is the *Ttn* gene, encoding TTN, a giant muscle structural protein which acts as a myofibrillar backbone attached to the Z-disk, the thin filament, the thick filament, and the M-band (Linke 2008). TTN regulates the passive stretch of cardiomyocytes via the I-band region of the protein which is comprised of sequential Ig, PEVK, and N2B domains (Linke 2008). In the heart, alternative splicing of *Ttn* results in a shorter ventricular isoform (N2B) and a longer atrial isoform (N2BA) (Freiburg *et al.* 2000). These two isoforms are coexpressed at different ratios during development as a mechanism for fine-tuning the passive stiffness of the cardiomyocyte (Cazorla *et al.* 2000). TTN acts as a signaling hub from which direct and indirect interactions with multiple proteins can lead to diverse cellular responses, including changes in gene expression (Linke 2008).

**Figure 5:**
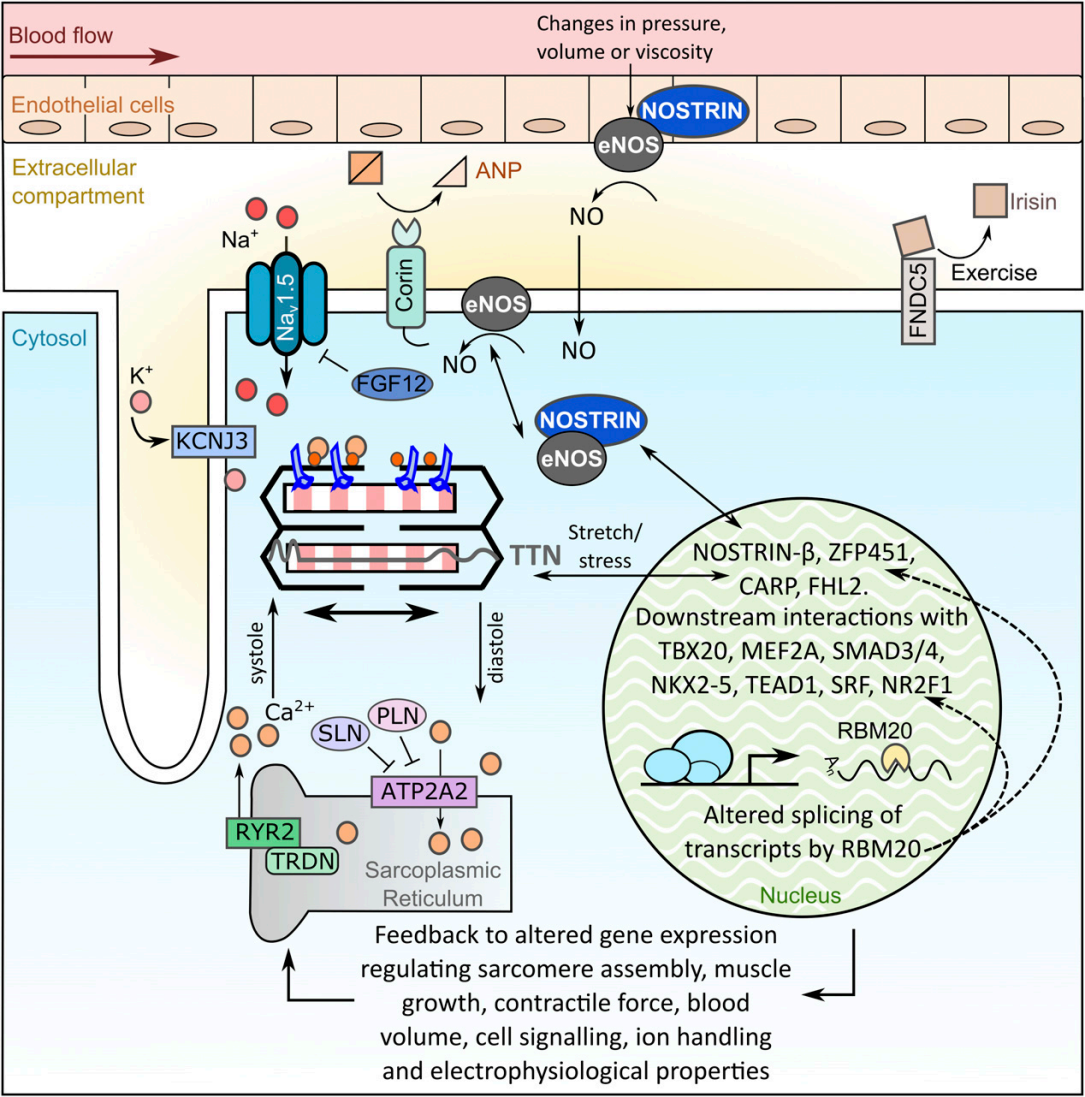
A model for pulmonary myocardium gene regulation in response to hemodynamic stress. Based on our gene network analysis we propose that hemodynamic changes trigger mechanosensory (stretch-stress) signaling from the sarcomere via TTN, and initiate NO signaling cascades which are transmitted to the myocyte nucleus via NOSTRIN. These signals initiate dynamic regulation of gene expression to enable fine-tuning of the contractile properties of the pulmonary myocardium. Gene expression changes may also be regulated at the post-transcriptional level by RBM20 which could feedback to regulate *Nostrin*, *Ttn*, and *Zfp451*. Collectively, these effects are predicted to result in altered contractile properties, ion handling, and hormonal signaling in the pulmonary myocardium to direct an effective response to the initial hemodynamic cue.

The pulmonary myocardium gene coexpression network (Table S3 in File S2) contains a number of genes coding for TTN-interacting proteins, including cardiac myosin-binding protein C (*Mybpc3*; one of the original 24 transcripts), α-actinin (*Actn2*), obscurin (*Obscn*), *Actc1*, cardiac ankyrin protein [*Ankrd1*; also known as cardiac ankyrin repeat protein (CARP)], myomesin (*Myom2*), and four and a half LIM domains 2 (*Fhl2*). Of these TTN-interacting proteins, FHL2 interacts with the N2B domain (present in both isoforms), while CARP interacts with the N2A domain (present only in the longer N2BA isoform) (Linke 2008). Both FHL2 and CARP have been shown to act as transcription factors in response to specific stimuli. For example, CARP is upregulated at the myofibril and in the nucleus in response to stretch (Miller *et al.* 2003), while FHL2 is upregulated in response to RhoA signaling (Philippar *et al.* 2004). FHL2 has also been shown to interact with SRF to antagonize expression of smooth muscle genes in differentiating embryonic stem cells (Philippar *et al.* 2004). Similarly, CARP overexpression in murine neonatal cardiomyocytes represses expression of *Anf*, *Actc1*, *Acta1*, β*MHC*, ventricular myosin light chain 2 (*Myl2*), and cardiac troponin C (*Tnnc*) (Mikhailov and Torrado 2008); genes which are associated with our pulmonary myocardium gene network. We therefore propose a model whereby mechanosensing by TTN could lead to altered gene expression of the pulmonary myocardium gene network via FHL2 or CARP (Figure 5), which might exert opposing transcriptional effects depending on the TTN isoform expressed (as CARP signaling would only be activated by the N2BA isoform). Interestingly, *Fhl2* had one of the highest fold-changes in expression in the left atria of patients with atrial fibrillation compared to healthy controls (Deshmukh *et al.* 2015), supporting a role for dysregulated TTN signaling in the pathobiology of atrial fibrillation.

Our eQTL analysis indicates that one of the genes potentially regulated by TTN is *Rbm20*, which in turn regulates *Ttn* isoform expression. RBM20 mediates alternative splicing of *Ttn*, resulting in increased expression of the shorter N2B isoform (increased stiffness) at the expense of the longer N2BA isoform (Beraldi *et al.* 2014; Guo *et al.* 2013; Wyles *et al.* 2016). RBM20 is also involved in alternative splicing of *Mef2a*, *Znf451*, and *Trdn* (Beraldi *et al.* 2014; Guo *et al.* 2013), and is associated with altered expression of *Nkx2-5*, *Myl4*, *Myl7*, *Tnnt2*, *Mybpc3*, and *Actc1* (Beraldi *et al.* 2014). These changes in gene expression could be due to alternative splicing of MEF2A, which is likely to regulate the pulmonary myocardium gene network (Table 4). In knockout/-down studies, loss of RBM20 is associated with altered splicing and expression levels of >200 genes, and the authors conclude that RBM20 underpins structural and functional development of cardiomyocytes (Beraldi *et al.* 2014). RBM20 is also likely to be an important regulator of the pulmonary myocardium molecular phenotype, is associated with NKX2-5 expression, and could be an important therapeutic target in atrial fibrillation.

As a whole, our data imply that regulatory polymorphisms which alter expression levels of upstream regulators of this gene network (such as *Nkx2-5*, *Mef2a*, *SRF*, *Nostrin*, *Znf451*, and *Ttn*) might predispose to atrial fibrillation. This hypothesis is supported by the observation that expression of *Ttn*, one of our candidate upstream regulators, is increased in atrial fibrillation patients with persistent disease (Deshmukh *et al.* 2015) and a recent GWAS that has for the first time identified the *TTN* locus as a susceptibility locus for atrial fibrillation in humans (Christophersen *et al.* 2017). Further, genes within our gene network that are regulated by the Chr2 eQTL containing *Ttn* are also dysregulated in atrial fibrillation (*Rbm20*, *Atp2a2*, *Fndc5*, and *Mybphl*) (Deshmukh *et al.* 2015) and dysregulation of transcription factors and ion/calcium handling genes could drive the switch from working myocardium to the pacemaker phenotype observed in the pulmonary myocardium in atrial fibrillation.

Of particular interest in our gene network is the expression of *Cacna2d2* which codes for the α-2/δ subunit of the voltage-dependent calcium channel complex, a subunit that is highly and predominantly expressed in the sinoatrial and atrioventricular nodes (Marionneau *et al.* 2005). This gene is expressed at low levels in our gene network but is regulated from a suggestive eQTL on Chr1: 33 (one of the three loci from which our gene network is coregulated). Since expression of this subunit is associated with nodal tissues, upregulation of *Cacna2d2*, subsequent to dysregulation of our gene network, could predispose to gain of pacemaker activity. In support of this, *Cacna2d2* expression is reported to be highly sensitive to dosage of the transcription factor TBX5 (Mori *et al.* 2006), another gene in our extended gene network. TBX5 is an essential factor for development of the cardiac conduction system including the atrioventricular bundle and bundle branches (Arnolds *et al.* 2012; Mori *et al.* 2006; Moskowitz *et al.* 2004). TBX5 has also been shown to interact and cooperate with both NKX2-5 (Hiroi *et al.* 2001; Puskaric *et al.* 2010) and GATA4 (Munshi *et al.* 2009) (also in our gene network) to drive commitment to a nodal cell phenotype (Stefanovic *et al.* 2014). Since NKX2-5 is known to suppress the nodal phenotype (Ye *et al.* 2015), a fine balance between TBX5 interactions with NKX2-5 *vs.* GATA4 may underlie the maintenance of the working myocardium in the pulmonary myocardium.

Taken together, a model is proposed whereby hemodynamic stress triggers mechanosensory (stretch-stress) signaling from the sarcomere and initiates NO signaling cascades from the adjacent pulmonary epithelia, which are transmitted back to the pulmonary myocardium myocyte nuclei (Figure 5). This leads to dynamic regulation of gene expression, enabling fine-tuning of the contractile properties of the pulmonary myocardium, such as the rate and force of contraction, as well as influencing blood volume, ion transport, and hormone signaling. Our findings indicate that genetic variability at these loci can modify levels of gene expression in the pulmonary myocardium, and could modify venous return to the left atrium as well as pathological features such as hypertrophy, fibrosis, and initiation of ectopic beats. Hence the genes/loci identified in this study could be linked to atrial fibrillation in humans triggered by pulmonary events. Indeed, these loci regulate genes that in humans are known to be associated with various heart diseases (dilated cardiomyopathy, hypertrophic cardiomyopathy, and arrhythmia) and are therefore viable candidates for involvement in the pathobiology of atrial fibrillation.

## Supplementary Material

Supplemental material is available online at www.g3journal.org/lookup/suppl/doi:10.1534/g3.117.044651/-/DC1.
