# Supplementary material for: Gene Expression Networks in the Murine Pulmonary Myocardium Provide Insight into the Pathobiology of Atrial Fibrillation
Source: G3 (Bethesda). 2017 Jul 18;7(9):2999–3017. doi: 10.1534/g3.117.044651 (PMC5592927; doi:10.1534/g3.117.044651)
Supplement: Supplementary file 1 [file 2999FileS1.docx]

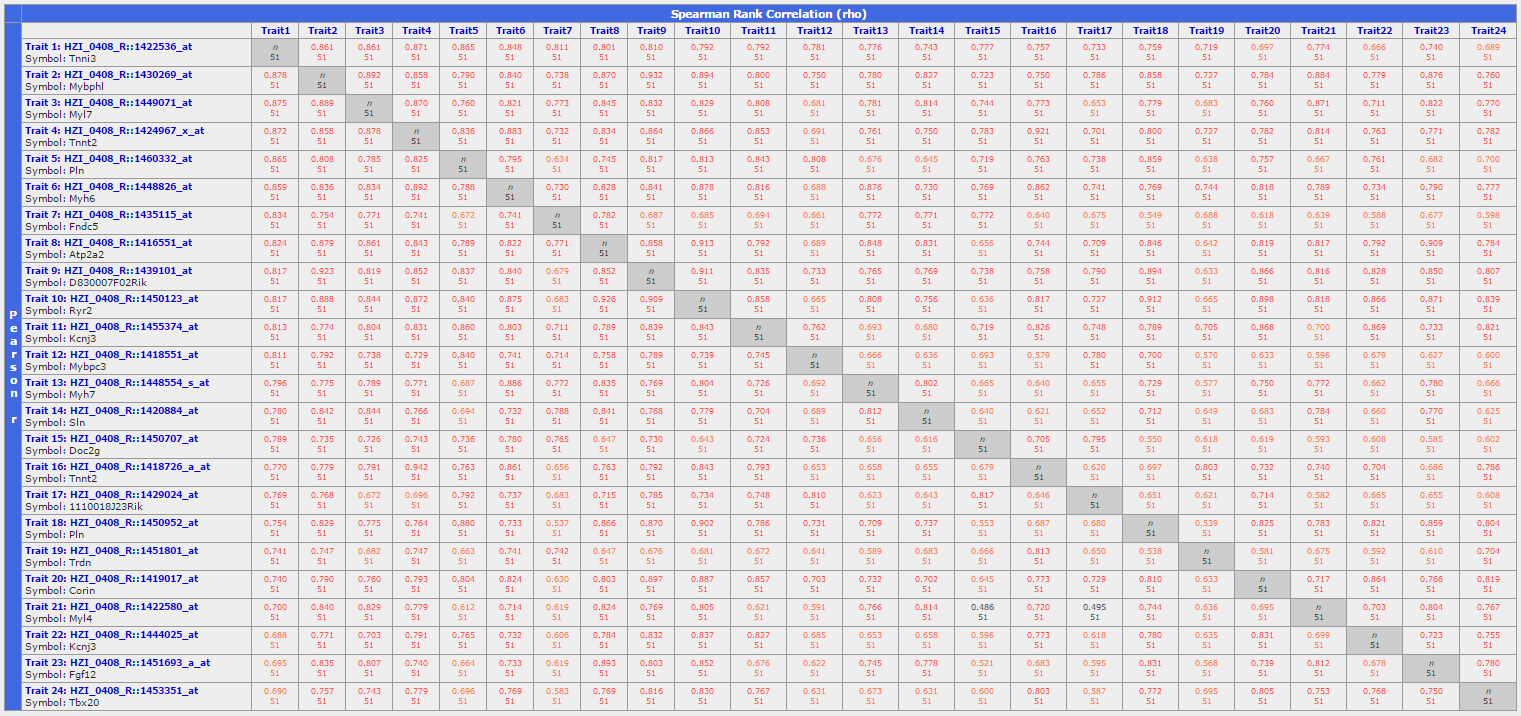


**Supplementary Figure 1. All 24 transcripts in the pulmonary myocardium gene network are positively correlated with each other.**

Pairwise comparison of all 24 transcripts was performed and all probe pairs were positively correlated. Pearson correlations (listed in the bottom left panel of the matrix) >0.7 are displayed in red; those between 0.5 and 0.7 in orange; those <0.5 in black.

**
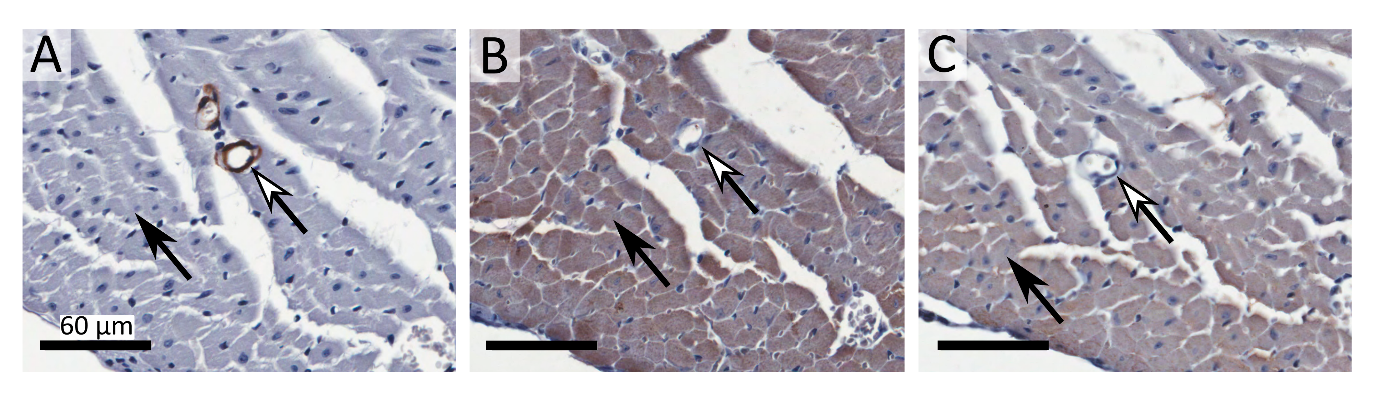
**

**Supplementary Figure 2. Cardiac troponin I (Tnni3) and smooth muscle** α-**actin (Acta2) are localised to different structures in mouse heart**.

Serial sections of cardiac ventricular tissue were immunostained for smooth muscle α-actin (**A**; Acta2), cardiac troponin I (**B**; Tnni3) and cardiac α-actin (**C**; Actc1). Distinct cell type localisation of Tnni3 in cardiomyocytes (black arrow) and Acta2 in the smooth muscle of small arteries (white arrow) are indicated. All scale bars are 60 μm.
